# A High-Resolution MRI Study of the Relationship Between Plaque Enhancement and Ischemic Stroke Events in Patients With Intracranial Atherosclerotic Stenosis

**DOI:** 10.3389/fneur.2018.01154

**Published:** 2019-01-08

**Authors:** ErLing Wang, Sai Shao, Shan Li, Peng Yan, YuanYuan Xiang, Xiang Wang, JiFeng Li, Guangbin Wang, QinJian Sun, YiFeng Du

**Affiliations:** ^1^Department of Neurology, Shandong Provincial Hospital affiliated to Shandong University, Jinan, China; ^2^Department of Radiology, Shandong Medical Imaging Research Institute, Jinan, China

**Keywords:** high-resolution MRI, intracranial atherosclerosis, ischemic stroke, plaque, stenosis

## Abstract

**Purpose:** To investigate the relationships among the degree of intracranial atherosclerotic stenosis (ICAS), plaque enhancement (PE), and ischemic stroke events (ISEs) using 3. 0 T high-resolution magnetic resonance imaging (HR-MRI).

**Materials and Methods:** Fifty-two ICAS patients who underwent HR-MRI were retrospectively analyzed. The patients were divided into two groups according to the results of whole-brain digital subtraction angiography (DSA): the mild-moderate stenosis group (group MID) and the severe stenosis group (group SEV). According to the onset time of the ISEs, the plaques were divided into the acute/sub-acute phase culprit plaque group (group ACU, within 1 month), the chronic-phase culprit plaque group (group CHR, more than 1 month), and the non-culprit plaque group (group NON). Two neuroradiologists independently measured the signal intensity of PE and pituitary enhancement in the HR-MRI and calculated the ratio of the two indices. According to the ratio, the patients were divided into three groups: the marked enhancement group (group MA), the mild enhancement group (group ME), and the no enhancement plaque group (group NO). The relationships among the degree of ICAS, the degree of PE and ISEs were analyzed.

**Results:** Seventy-two ICAS plaques were identified in 52 patients. The multiple independent samples Kruskal-Wallis H test showed that the differences among group ACU, CHR, and NON were significant in the degree of PE (*P* = 0.002). Group CHR and group NON were combined as the non-acute phase group (group non-ACU). Group NO and group ME were combined as the non-marked enhancement group (group non-MA). The comparison between group ACU and group non-ACU showed significant differences in the degree of both ICAS (*P* = 0.014) and PE (*P* = 0.006) according to the univariate logistic regression. The multivariate logistic regression model was used to analyze the impact of the degree of ICAS and PE on ISEs, and the results showed that severe stenosis (*P* = 0.036) and marked PE (*P* = 0.013) were independent risk factors for acute ISEs, respectively.

**Conclusion:** Severe intracranial arterial stenosis and marked plaque enhancement are independent risk factors for acute ischemic stroke events, respectively. The study provides new ideas for further exploring the pathogenesis of stroke caused by intracranial atherosclerotic stenosis.

## Introduction

Intracranial atherosclerotic disease (ICAD) is the leading cause of global ischemic stroke (1). As an emerging imaging technology, high-resolution magnetic resonance imaging (HR-MRI) can not only clearly show the components of carotid plaques (2, 3) but can also show the lipids, fibrous cap, and calcification of intracranial arterial plaques, which have good consistency with pathological findings (4–7); thus, HR-MRI has become an ideal means to display intracranial arterial plaques and inner components (4, 8–10). An increasing number of HR-MRI studies have focused on the relationship between the characteristics of intracranial plaques and clinical symptoms. A study published in 2017 showed that, in patients with mild basal arterial stenosis, the plaques that caused symptomatic pontine infarctions were mostly distributed in the dorsal and bilateral walls of the basilar artery (11). Certain autopsy results have shown that symptomatic intracranial atherosclerotic stenosis (ICAS) caused by atherosclerotic plaques, the percentage of lipid nuclear within plaques, and neovascularization were significantly related to the occurrence of ischemic stroke (12). Currently, studies have shown that the degree of PE in patients with moderate-severe intracranial arterial stenosis is significantly related to ischemic stroke (13, 14). In this study, patients with mild-moderate and severe ICAS were included to further investigate the relationships among the degree of ICAS, the degree of PE and acute ischemic stroke events (ISEs) respectively.

## Materials and Methods

### Subjects

**Figure 1:**
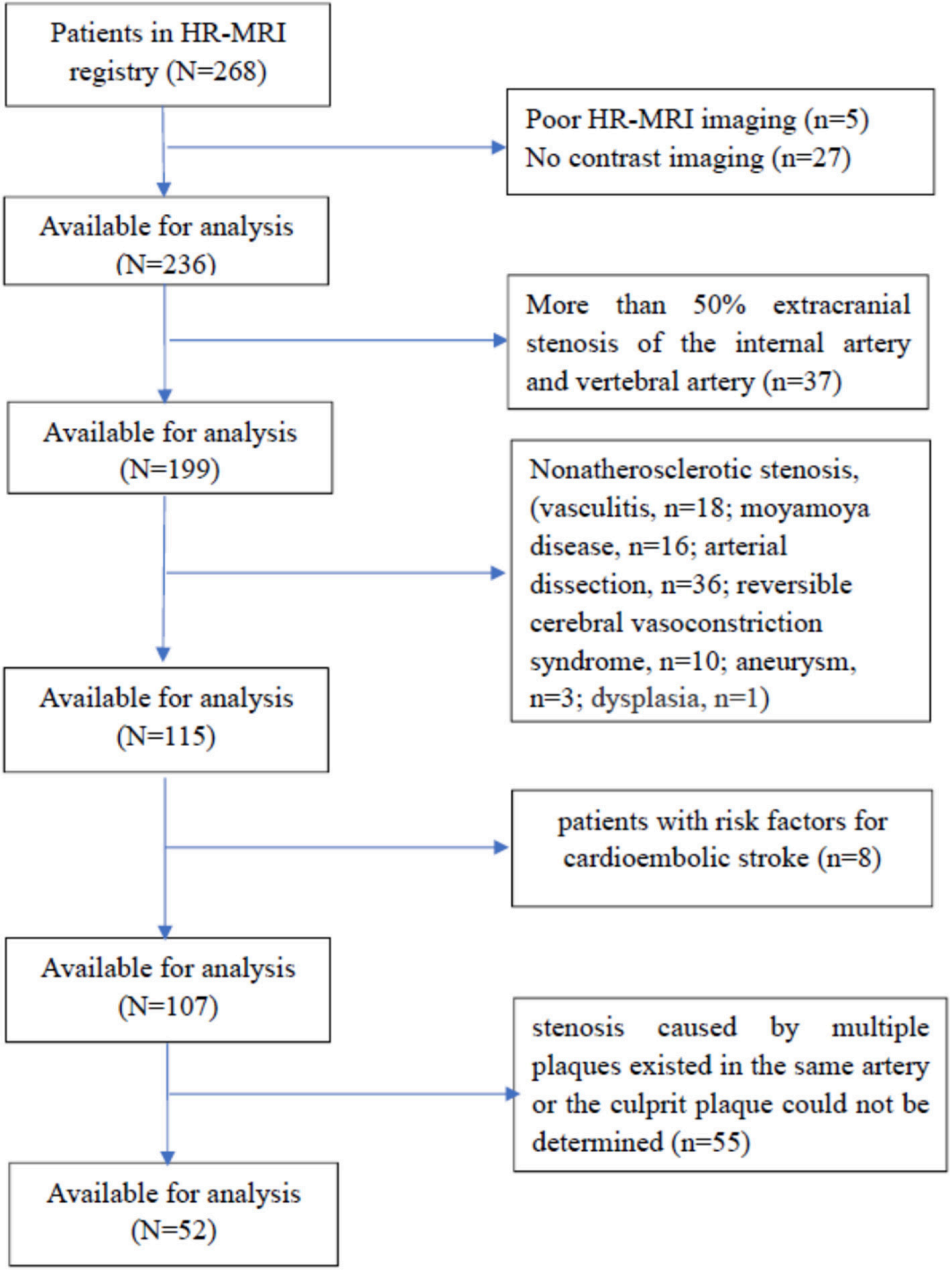
The flow chart for the inclusion of patients. HR-MRI, high-field and high-resolution magnetic resonance imaging.

We retrospectively analyzed the clinical data of 268 consecutive patients who were admitted to the Department of Neurology, Provincial Hospital Affiliated to Shandong University and who underwent intracranial arterial HR-MRI from January 01, 2015 to December 31, 2016. Fifty-two patients who met the criteria of internal atherosclerotic stenosis were enrolled. These patients had (1) confirmed ICAS by digital subtraction angiography (DSA) (including the intracranial segment of the internal carotid artery, middle cerebral artery, anterior cerebral artery, intracranial vertebral artery, basilar artery, and posterior cerebral artery), but the degree of stenosis was not limited (the degree of stenosis is >0% and <100%); and (2) HR-MRI-confirmed arterial stenosis caused by atherosclerotic plaques. Exclusion criteria consisted of (1) patients with an allergy to contrast agents; (2) patients with more than 50% extracranial stenosis of the internal carotid artery and vertebral artery shown in DSA, which might lead to intracranial hypoperfusion or collateral circulation and make more difficult the estimation of the intracranial stenosis; (3) patients with intracranial arterial stenosis caused by non-atherosclerotic entities according to the HR-MRI findings (such as vasculitis, moyamoya disease, arterial dissection, reversible cerebral vasoconstriction syndrome, aneurysm, or dysplasia); (4) patients lacking risk factors for atherosclerosis, such as hypertension, diabetes, dyslipidaemia, heart disease, obesity, smoking, or hyperhomocysteinemia; (5) patients with risk factors for cardioembolic stroke, such as atrial fibrillation, acute myocardial infarction, valvular heart disease, cardiomyopathy, or patent foramen ovale; and (6) patient-related causes of poor HR-MRI imaging. The flow chart for the inclusion of patients is shown in Figure 1. Approval for the study was obtained from the Ethics Committee of Provincial Hospital Affiliated to Shandong University (NO. 2017573). The study was carried out in accordance with The Code of Ethics of the World Medical Association (Declaration of Helsinki). Written informed consent was obtained from all the patients.

### MRI Parameters

All brain MRI examinations were performed on a 3.0 T MRI scanner (Achieve; Philips Medical Systems, Best, The Netherlands) with a parallel head imaging coil. The scan sequences included 3D T1 variable refocusing flip angle volume isotropic turbo spin-echo acquisition (VISTA), 3D-T2 VISTA, MRA, and T1 VISTA enhancement sequences.

The T1 VISTA scanning parameters were as follows: time of repetition (TR)/time of echo (TE), 800 ms/18 ms; echo column length (ETL), 16; field of view (FOV), 200 mm × 180 mm × 40 mm; layer thickness (Thk), 0.3 mm; matrix, 332 × 302 (isotropic spatial resolution: 0.3 × 0.3 × 0.3 mm); average, 1–2; parallel imaging (SENSE) factor, 2 (along the phase encoding direction); and scanning time, 378 s. Sensitized blood flow compensation was used to suppress the intraluminal blood signals, and a 90° refocus flip angle was used to enhance the air flow effect and to reduce images.

The T2 VISTA scanning parameters were as follows: TR/TE, 2,500 ms/231 ms; ETL, 16; FOV, 200 mm × 180 mm × 90 mm; layer thickness, 0.5 mm; matrix, 332 × 299 (isotropic spatial resolution: 0.5 × 0.5 × 0.5 mm); average, 1–2; parallel imaging (SENSE) factor, 2 (along the phase encoding direction); and scanning time, 398 s. Sensitized blood flow compensation was used to suppress the intraluminal blood signals, and a 90° refocus flip angle was used to enhance the air flow effect and to reduce images. The parameters of the enhanced T1 VISTA sequence were the same as those for the T1 VISTA sequence.

### MR Image Analysis

The atherosclerotic plaques in the HR-MRI were interpreted and measured by two neuroradiologists (Guangbin Wang, Sai Shao; 7 and 4 years of experience in plaque imaging, respectively, with extensive HR-MRI reading experience and blinded to the clinical data). The cross-sectional images of arterial vessels were selected to analyze the plaque characteristics.

The following method was used to quantify the degree of PE: Two independent neuroradiologists qualitatively graded plaque contrast enhancement based on its signal intensity on post-contrast HR-MRI by using the corresponding pre-contrast series for reference. The neuroradiologists were blinded to the characteristics of the study population, including brain MRI findings and clinical presentations. PE was graded by using a previously established grading scheme (14), and the patients were divided into three groups according to the ratio of the method, as follows: the no enhancement plaque group (group NO, indicating enhancement similar to or less than that of intracranial arterial walls without plaque in the same individual), the mild enhancement group (group ME, indicating enhancement greater than that of group NO but less than that of the pituitary infundibulum), and the marked enhancement group (group MA, indicating enhancement similar to or greater than that of the infundibulum). Cases in which neuroradiologists disagreed were reviewed together and resolved by consensus.

The DSA results were interpreted by two experienced neurointerventional physicians. ICAS was classified as either mild-moderate stenosis (<70%) or severe stenosis (≥70%). Multiple stenoses in one patient were recorded in detail. The two neurointerventional physicians were provided with each patient's medical history data and MRI data (including the T1 sequence, T2 sequence, FLAIR sequence, DWI sequence, and ADC sequence).The culprit plaque was identified based on the clinical presentation of the patient and clinical judgment of two neurologists. Acute ISEs included new infarction or transient ischaemic attacks. According to the onset time of the ischemic stroke event, the plaques were divided into the acute/subacute phase culprit plaques (group ACU, the plaque caused an ischemic stroke event within 1 month, followed by HR-MRI), the chronic-phase culprit plaques (group CHR, the plaque caused an ischemic stroke event more than 1 month, followed by HR-MRI), and the non-culprit plaques (group NON, the plaque did not caused an ischemic stroke event). When the two neurologists came to different conclusions, they negotiated to reach a consensus. When stenosis caused by multiple plaques existed in the same artery or the culprit plaque could not be determined due to other reasons, the plaque was excluded. The neurologists who determined culprit plaques were blinded to features of plaques in the HR-MRI.

### Statistical Analyses

All data analyses were conducted using the SPSS 20.0 software package (SPSS, Inc., USA). The measurement data are expressed as mean ± standard deviation (SD). The multiple independent samples Kruskal-Wallis H test was used to analyze the degree of PE among group ACU, CHR, and NON. The relationships among the degree of ICAS, the degree of PE and ISEs were analyzed by the univariate logistic regression. Variables were included for multivariate analysis if they were *P* < 0.05 in the univariate analysis. A *P* < 0.05 was considered as statistically significant.

## Results

**Figure 2:**
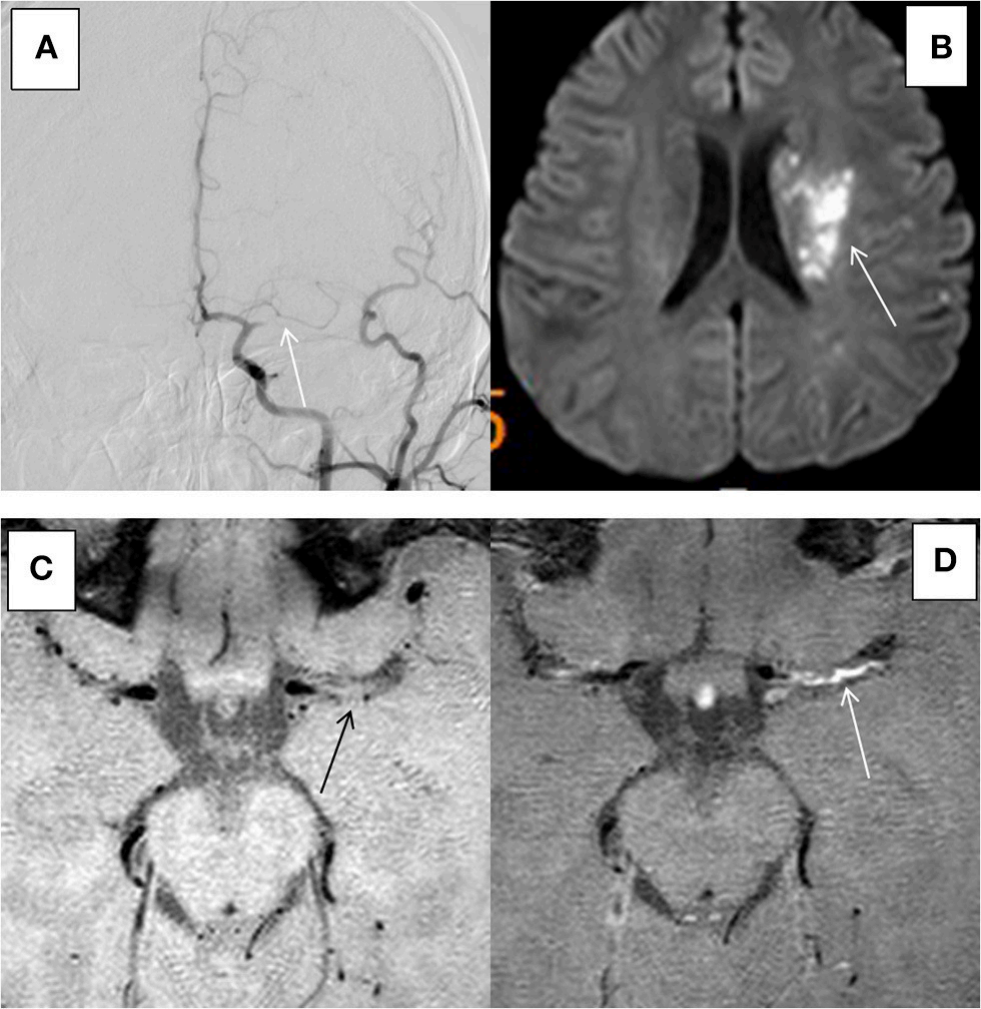
HR-MRI of a culprit MCA occlusion in an 28-years-old male with a history of previous hypertension. **(A)** DSA showed occlusion of the left MCA. **(B)** DWI showed that the left side of the paraventricular was limited by diffusion. **(C)** HR-MRI showed plaque formation in the Ml segment of MCA. **(D)** High-resolution enhanced Tl WI showed marked enhancement of the Ml segment of MCA.

Fifty-two patients met the inclusion and exclusion criteria, with the average age of 59.2 ± 10.8 years (ranging from 28 to 78 years), including 36 males (69.2%). All the patients had ≥1 vascular risk factor(s), and 34 patients (65.4%) had 2 or more vascular risk factors. The most common risk factors were hypertension, diabetes, smoking, and hyperlipidaemia. The demographic and clinical characteristics of group ACU and group non-ACU (by combining group CHR and group NON) were shown in Table 1. The youngest patient was only 28 years old and had a history of hypertension and poor blood pressure control. DSA showed occlusion of the left middle cerebral artery in this patient, and MRI showed an acute/subacute cerebral infarction in the left semi-oval center, temporal lobe, and basal ganglia region. High-resolution enhanced T1WI showed marked enhancement of the M1 segment of MCA (Figure 2).

**Table 1:** Demographic and clinical characteristics of group ACU and group non-ACU (by combining group CHR and Group NON).

	**Group ACU(*n* = 31)**	**Group non-ACU(*n* = 21)**	***P***
Male (%)	21 (67.7%)	15 (71.4%)	0.777
Age (years)	61.06 ± 11.90	56.38 ± 8.43	0.126
Smoker (%)	10	10	0.264
Alcoholism (%)	13	9	0.947
Hypertension (%)	25	16	0.700
Diabetes (%)	10	10	0.264
Blood glucose (mmol/L)	6.28 ± 2.16	6.71 ± 2.15	0.502
HbA1c (%)	16.20 ± 4.10	15.49 ± 3.41	0.535
Total cholesterol (mmol/L)	4.10 ± 0.95	4.09 ± 0.99	0.962
HDL (mmol/L)	1.05 ± 0.26	1.00 ± 0.18	0.467
LDL (mmol/L)	2.54 ± 0.70	2.62 ± 0.81	0.725
Triglycerides (mmol/L)	1.83 ± 1.67	1.90 ± 0.90	0.868
Homocysteine (mmol/L) (mmol/L)	12.88 ± 8.52	12.83 ± 5.22	0.982
WBC (/L)	6.94 ± 2.41	6.88 ± 1.48	0.924
Neutrophil percentage (%)	61.94 ± 8.19	61.07 ± 8.20	0.737

*The P-values for male, smoking, alcohol consumption, hypertension, and diabetes are obtained by using chi-square test. The P-values for other characteristics are obtained by two independent samples mean t-test. ACU, acute; CHR, chronic; NON, non-culprit; HbA1c, hemoglobinA1C; HDL, high-density lipoprotein; LDL, low-density lipoprotein; WBC, white blood cell count. Data are presented as number (%) or mean ± SD*.

Seventy-two intracranial atherosclerotic plaques were identified in 52 patients, Among these plaques, 18 (25%) resulted in mild-moderate arterial stenosis, while 54 (75%) resulted in severe arterial stenosis. According to the degree of enhancement, there were 8, 12, and 52 plaques in group NO, group ME, and group MA, respectively. According to the presence or absence of ISEs and the time interval between the HR-MRI examination and the onset of ISEs, there were 31, 13, and 28 plaques in group ACU, group CHR, and group NON, respectively.

**Figure 3:**
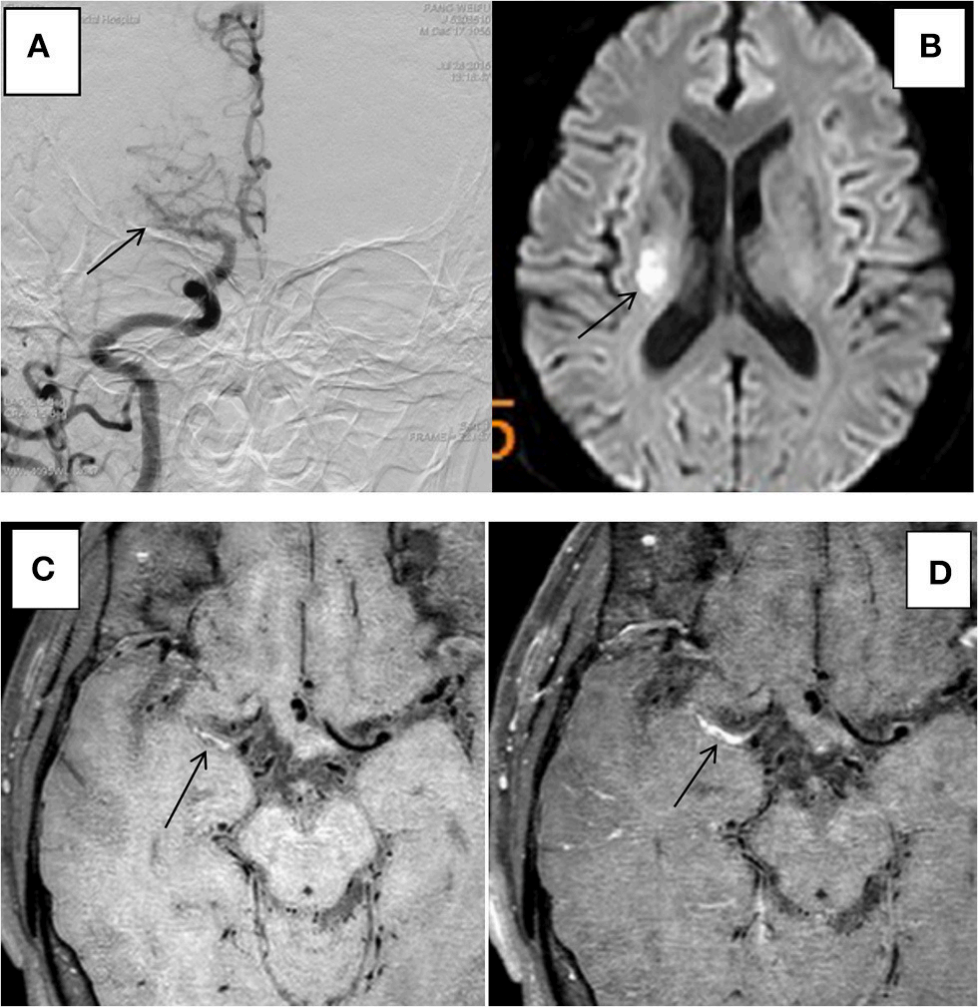
HR-MRI of a culprit MCA occlusion in an 59-years-oldmale. **(A)** DSA showed occlusion of the right MCA. **(B)** DWI showed that the right half of the oval center was limited by diffusion. **(C)** HR-MRI Tl WI showed plaque formation in the Ml segment of MCA. **(D)** High-resolution enhanced Tl WI showed marked enhancement of the Ml segment of MCA.

**Figure 4:**
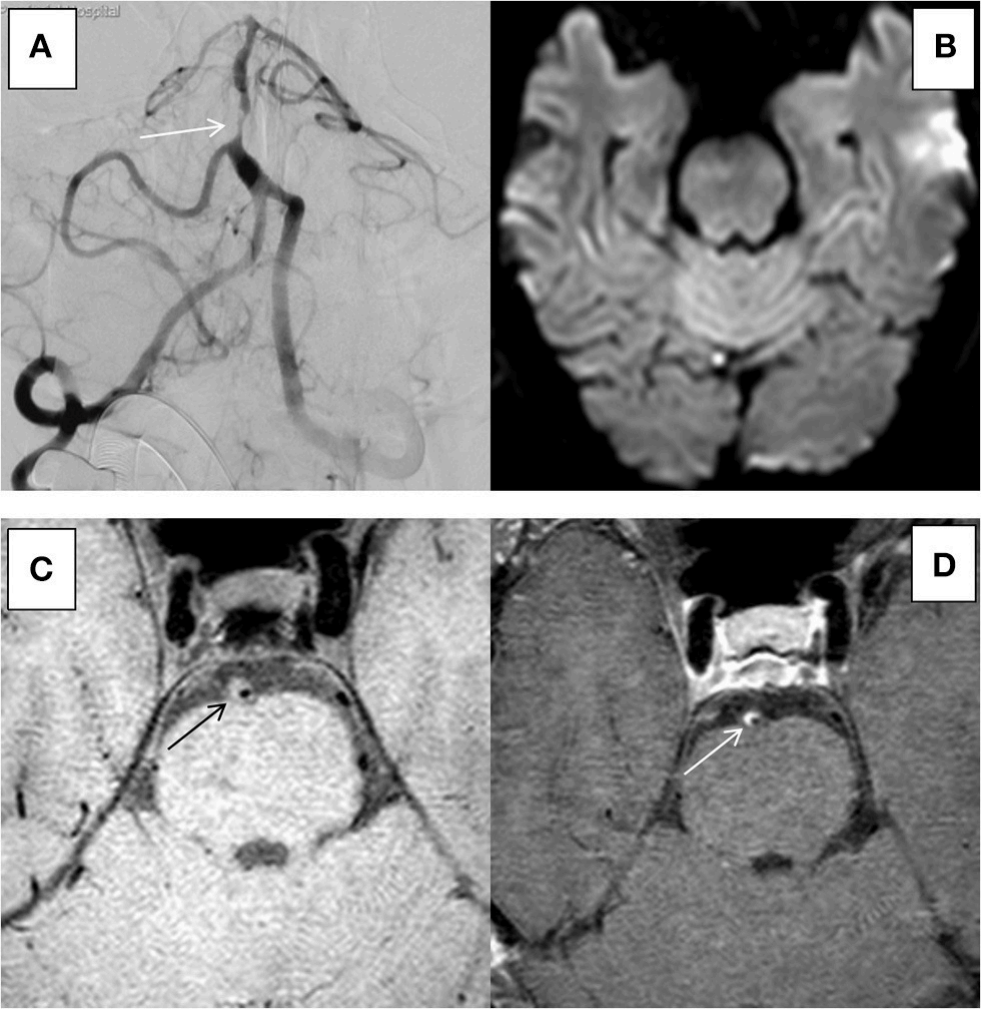
HR-MRI of an non-culprit BA severe stenosis in an 55-years-old male. **(A)** DSA showed severe stenosis of BA. **(B)** DWI showed that there was no limitation by diffusion. **(C)** HR-MRI Tl WI showed plaque formation in BA. **(D)** High-resolution enhanced Tl WI showed marked enhancement of BA.

The 31 plaques in group ACU all showed enhancement, with 3 plaques having mild enhancement (9.7%) and 28 plaques having marked enhancement (90.3%) (Figure 3). All 13 plaques in group CHR showed enhancement, with 3 plaques having mild enhancement (23.1%) and 10 plaques having marked enhancement (76.9%). The 20 plaques in group NON showed enhancement, with 6 plaques having mild enhancement (21.4%), 14 plaques having marked enhancement (50%) (Figure 4), and 8 plaques demonstrating non-enhancement (28.6%). Among the 72 plaques, 8 plaques showed no enhancement, all of which were non-culprit plaques. The distribution is shown in Table 2.

**Table 2:** Distribution characteristics of plaque of patients.

	**Non-enhancement (%)**	**Mild enhancement (%)**	**Marked enhancement (%)**
Group ACU	0	3 (9.7%)	28 (90.3%)
Group CHR	0	3 (23.1%)	10 (76.9%)
Group NON	8 (28.6%)	6 (21.4%)	14 (50%)

*Group ACU, the acute/sub-acute phase culprit plaque group; Group CHR, the chronic-phase culprit plaque group; Group NON, the non-culprit plaque group*.

The multiple independent samples Kruskal-Wallis H test showed significant differences in the degree of PE among group ACU, group CHR, and group NON (*P* = 0.002) Group CHR and group NON were combined as the non-acute phase group (group non-ACU). Group NO and group ME were combined as the non-marked enhancement (group non-MA). The comparisons between group ACU and group non-ACU showed significant differences in the degree of both ICAS (*P* = 0.014) and PE (*P* = 0.006) according to the univariate logistic regression. The multivariate logistic regression model was used to analyze the impact of arterial stenosis and PE on the ISEs in the acute phase, and the results showed that severe stenosis (*P* = 0.036, OR 4.5, 95%CI 1.1–18.3) and marked PE (*P* = 0.013, OR 5.7, 95%CI 1.4–22.7) were independent risk factors for acute ISEs, respectively (Table 3).

**Table 3:** Association of acute/sub-acute culprit plaques with arterial stenosis and plaque enhancement.

			**Univariate**		**Multivariate**	
	**No. of plaques**	**No. of acute/sub-acute culprit**	**OR(95%CI)**	***P*-value**	**OR (95%CI)**	***P*-value**
**STENOSIS**						
Group MID	18	3	1.0		1.0	
Group SEV	54	28	5.4 (1.4, 20.8)	0.014	4.5 (1.1, 18.3)	0.036
**ENHANCEMENT**						
Group Non-MA	20	3	1.0		1.0	
Group MA	52	28	6.6 (1.7, 25.3)	0.006	5.7 (1.4, 22.7)	0.013

*Group MID, the mild-moderate stenosis group; Group SEV, the severe stenosis group; Gruop non-MA, the non-marked enhancement group; Group MA, the marked enhancement group*.

## Discussion

The primary age group of ICAD in this study ranged from 50 to 69 years old. However, the youngest patient was only 28 years old. The patient was included in the study due to the ischemic stroke being caused by cerebral atherosclerosis based on the indices of risk factors, clinical manifestations, DSA, and HR-MRI. This case suggested that young patients should not be eliminated the diagnosis of atherosclerotic disease.

The most common risk factors of ICAD were hypertension, smoking, diabetes, and hyperlipidaemia. Forty-one of the 52 (78.8%) patients enrolled in the study had a history of hypertension; 20 (38.5%) patients suffered from diabetes; and 20 (51.4%) patients had a history of smoking (all male). The proportion of hyperlipidaemia was 32.7%.

In this study, there were 8 non-enhanced plaques, all of which were non-culprit plaques. The culprit plaques (acute/subacute plaques and chronic plaques) have different degrees of PE. A study by Wasserman in 2014 in Radiology and other study in 2010 in Atherosclerosis also found similar phenomena (14, 15). We speculated that this phenomenon indicated that non-enhanced plaque might be stable. Some studies have shown that plaques were marked enhancement within 4 weeks of stroke onset. As time progressed, the degree of PE began to gradually decline (16), and the duration of PE lasted longer and generally exceeded the acute phase. In this study, it was observed that some of the patients in group CHR also showed marked enhancement, which was consistent with this view. However, longitudinal studies are still needed to further elucidate the evolution of plaque intensity as time progressed and the relationship between the evolution of PE and clinical symptoms.

Studies have shown that extracranial PE was associated with increased neovascularization and endothelial permeability, both of which could promote the penetration of contrast agents into plaques (17–21), and the extent of carotid PE has a strong correlation with ischemic stroke (22). However, the pathological mechanism of intracranial PE remains unclear. A previous study (23) published in 2006 by Amarenco has shown that there was no clear correlations between intracranial PE and ISEs, but the sample size was too small. Currently, some studies have focused on the relationship between the degree of intracranial arterial PE and ISEs. Some of which has shown strong correlations between PE and ISEs (13, 14, 16, 24, 25). Unfortunately, most of these studies have only included more than 50% of arterial stenosis, which is one of the mechanisms of acute ISEs, moreover, without the simultaneous analysis of the correlations between the degree of arterial stenosis and acute ISEs. Therefore, the results of these studies could not fully understood whether acute stroke was associated only with arterial stenosis or with PE. A recent study (26) published in 2017 by Chen has shown that there was no association between culprit nor non-culprit plaques and enhancement, still no stenosis stratified. Therefore, few studies have simultaneously analyzed the relationships among arterial stenosis, PE and ISEs. This study bridges the gap. In this study, the univariate and multivariate analysis showed a high correlation between marked PE and ISEs after adjustment for the effect of arterial stenosis, and there was a strong association between severe intracranial arteries stenosis and ISEs after adjustment for the effect of enhancement. Therefore, severe intracranial arterial stenosis and marked PE were independent risk factors for acute ISEs, which suggests that severe stenosis and marked PE play important roles on the development of acute ISEs, respectively.

This study has several highlights. First, most of the previous similar studies only analyzed the relationship between PE and ISEs without the consideration of the effect of arterial stenosis degree on ISEs. This study made up for the insufficience. This study showed that the potential confounder of arterial stenosis cannot be ignored when studying the relationship between PE and ISEs. Secondly, the study further confirmed the correlation between intracranial arterial stenosis and ISEs, which might provide a reference for the treatment of patients with severe intracranial arterial stenosis. Therefore, the study will benefit in improving risk stratification of patients with ICAS and making treatment decision for individual patients.

There were several limitations in this study. First, because there were fewer samples of group NO and group ME, the two groups were merged into group non-MA in multivariate analysis. If the sample size was sufficient, we can perform a subgroup analysis of the degree of PE or stenosis, the study may obtain more detailed results. Secondly, the current study was cross-sectional and failed to explore longitudinal changes in the degree of enhancement of the same plaque as time progressed. Therefore, further studies with larger sample sizes will be beneficial for establishing the relationships among the degree of arterial stenosis, PE and ISEs. Thirdly, the intracranial arteries of the same patient were not fully included due to the limitations of the scan planes, which may have resulted in sample loss and affected the results.

## Conclusion

Severe intracranial arterial stenosis and marked plaque enhancement are independent risk factors for acute ischemic stroke events, respectively. The study provides new ideas for further exploring the pathogenesis of stroke caused by intracranial atherosclerotic stenosis.

## Ethics Statement

This study was carried out in accordance with the recommendations of the Ethics Committee of Shandong Provincial Hospital Affiliated to Shandong University with written informed consent from all subjects. All subjects gave written informed consent in accordance with the Declaration of Helsinki. The protocol was approved by the Ethics Committee of Shandong Provincial Hospital Affiliated to Shandong University name of committee.

## Author Contributions

QS and EW: Study concept and design, analysis and interpretation, critical revision of the manuscript for important intellectual content; SS and GW: Image analysis, acquisition of data, critical revision of the manuscript for important intellectual content; SL, XW, PY, and YX: Analysis and interpretation, critical revision of the manuscript for important intellectual content; JL and YD: Study supervision, critical revision of the manuscript for important intellectual content.

### Conflict of Interest Statement

The authors declare that the research was conducted in the absence of any commercial or financial relationships that could be construed as a potential conflict of interest.
